# Relationship of Emotional Intelligence with academic scores and gender in students of Masters in Health Professions Education (MHPE) at a Public Sector University

**DOI:** 10.12669/pjms.39.6.7399

**Published:** 2023

**Authors:** Afzal Fatima, Syeda Kauser Ali

**Affiliations:** 1 Afzal Fatima, MBBS, DGO, MHPE Senior Lecturer, Directorate of Educational Development, Ziauddin University, Karachi, Pakistan; 2 Syeda Kauser Ali, MBBS, MHPE, PHD Chairperson, Institute of Medical Education, Jinnah Sindh Medical University, Karachi, Pakistan

**Keywords:** Emotional intelligence, Academic scores, Health profession educationists

## Abstract

**Objective:**

To determine the correlation between Emotional Intelligence (EI) scores and academic scores and identify other factors that have a relationship with EI.

**Methods:**

Cross-sectional analytical study was conducted in the year 2021 on 52 students enrolled in three batches of the MHPE program of a public sector university in Karachi. Data was collected by using Sterrett’s Emotional Quotient Self-Assessment Checklist along with a self-constructed form for obtaining demographic profile information. Descriptive analysis was done by calculating percentage, mean and standard deviation. The correlation was done by using Spearman rho and association was tested by Chi-square.

**Results:**

Data from forty-one participants showed that the majority need to improve their EI. Females and married participants had higher EI scores. There was a significant positive correlation between EI scores and academic performance but no significant association was found between EI and gender of the participants.

**Conclusion:**

The study showed that EI competencies have a strong positive correlation with the academic performance of MHPE students. Hence, it emphasizes the need for including development of EI in the outcomes and curricula of the existing MHPE program.

## INTRODUCTION

Emotional intelligence (EI) is a category of intelligence that comprises the ability of a person to regulate his or her own and others’ emotions, to differentiate between them and to use the information to manage their thoughts and activities.^1^ There has been an increasing trend to determine and improve the EI levels in various professions as the overall organizations’ behaviour and outcomes could be affected by it.^2^

The healthcare system is moving towards multidisciplinary care and EI skills are needed to improve interpersonal communication skills and managing conflicts between stakeholders.^3^ A professional culture is necessary in the medical industry to ensure high-quality patient care, advance moral behaviour, and promote mutual respect between patients and healthcare professionals.^3^

There is a close relation between emotional intelligence and medical education competencies and it is important that EI can be developed and improved as an integral part of training programs.^4^ Studies have shown that healthcare employees with high emotional stability had greater level of job satisfaction.^5^ Good academic performance has been considered to depend upon high intelligence quotient (IQ) however studies have shown that emotional values are more essential than the cognitive ones for a successful professional career.^6^

The Master of Health Professions Education (MHPE) programs prepare students to be competent teachers, scholars, leaders and innovators who would improve the education system of healthcare professionals and in turn future of health care. The graduates of these programs aspire to be change agents for improvement in undergraduate and postgraduate health professionals’ educational programs, hence having EI is essential to their role.

One of the major roles of health professions educationists is initiating and implementing the required changes in the curriculum, teaching learning and assessment policies, processes and procedures. This is done by conducting faculty development activities geared towards improving the quality of education and better healthcare.^7^

It has been noted that the current MHPE curricula do not have formal course on EI. The study aimed to determine the level of EI of the MHPE students and its relationship with gender and academic achievement in a public sector University of Karachi.

## METHODS

A cross-sectional study was conducted at a public sector University of Karachi in 2021. The program is a 2.5-year, blended program which emphasizes the development of critical thinking and professional skills that an educationist will need once s/he enters this profession. Universal sampling was done and all students (n=52) enrolled in three batches of the MHPE program offered by the University were included in the study. Eight students did not give consent for participation and the performance score of three students was not available, so they were excluded from the study. The final sample consisted of 41 participants.

Emotional Intelligence scores were collected by administering Sterrett’s Emotional Quotient Self-Assessment Checklist (SEQSAC) that is freely available for use.^8^ It has six domains, each having five statements. respectively, making a total of 30 statements. This questionnaire was pre-validated and tested for reliability (Cronbach’s alpha=0.82).^9^

Participants were asked to rate each numbered statement in the SEQSAC using a 5-point Likert scale, 1=Never and 5=Always. Ratings were added for each category to obtain a total for that specific domain of EI. The minimum score is five and the maximum 25 respectively for each domain. For good Emotional Intelligence, a value of 20 and above has been reported to be standard in each domain. Any score below this identifies a need for improvement in the respective area.^8^ The total EI score is the sum of all 6-domain scores.

The maximum and minimum total scores of the questionnaire (30 questions) are 150 and 30 respectively. For good Emotional Intelligence a value of 120 and above is acceptable, any score below this value needs improvement in the EI.^10^

### Ethical Approval

Ethics approval was taken from relevant institutional bodies (20^th^ June 2021, Ref. No. JSMU/BASR/2021/-60). The Sterrett’s EI questionnaire was administered online, which the participants could access after giving their consent and demographic information.

The completed questionnaires were submitted to the Institute of Medical Education (IME) for assigning codes to the respondents and their respective score sheets of Modules one and two of the MHPE program to ensure anonymity. Data were analyzed using SPSS-24. Descriptive data is presented as mean and standard deviation of total EI score, six domain scores and age. Percentages were calculated for gender and marital status. Spearman Rho was used to test the correlation between EI and academic scores and Chi-square test was used for the association between EI and gender. A ’ p-value of < 0.05 was taken as significant.

## RESULTS

Forty-one participants were included in the study. Among them, 34 (82.92%) were females and seven (17.1%) were males. The mean age of the participants was 41 years. The reliability of SEQSAC in this study was α = 0.90. For descriptive analysis, the age of the participants was divided into two groups. Group-1, <35 years and Group-2, ≥ 35 years. There were 37 (90.2%) married participants while 04 (9.8%) were single.

The mean EI score of the participants was 112. Among 41 participants, 11 (26.80%) had good EI scores while 30 (73.20%) needed improvement. The highest EI score was 136 and the lowest was 87. More females had a higher EI score (24.4%) than males (2.4%). The majority of participants from age Group-2 (19.51%) had good EI scores (>120) and from Group-1 only 7.31% of participants scored “good”. The EI scores were found to be more in married (24.39%) than in single participants (2.44%) (Table-I).

**Table-I:** Descriptive analysis.

Variable	Mean	Standard Deviation	Minimum	Maximum	EI scores Good ≥120		EI scores need improvement <120	

					N	%	N	%
EI	112.63	12.951	87	136	11	26.8	30	73.2
Age (year)	41.00	9.758	28yr	65yr	-	-	-	-
Group-1 <35yrs. (12=29.26%)	-	-	-	-	3	7.31	9	21.95
Group-2 ≥35yrs. (29=70.73%)	-	-	-	-	8	19.51	21	51.21
Variable	-	-	N	%	N	%	N	%
Gender	-	-	41	-	-	-	-	-
Male	-	-	7	17.1	1	2.4	6	14.6
Female	-	-	34	82.92	10	24.4	24	58.5
Marital Status	-	-	41	-	-	-	-	-
Married	-	-	37	90.2	10	24.39	27	65.85
Single	-	-	4	9.8	1	2.44	3	7.31

Participants scored more in the domains of Motivation and Empathy respectively followed by Self-awareness and Self-confidence. They scored least in the domains of Self-control and Social-competence. The majority of participants scored in the category of need improvement in all the domains (Table-II).

**Table-II:** Mean scores of six domains and number of participants in each EI category

S. No	EI Domains	Mean	Standard Deviation	Minimum	Maximum	Good: ≥20		Need Improvement: < 20	

						N	%	N	%
1	Self-awareness	18.93	2.500	12	24	17	41.5%	24	58.5%
2	Self-confidence	18.49	2.993	12	23	18	43.9%	23	56.1%
3	Self-control	18.22	2.151	14	22	14	34.1%	27	65.9%
4	Empathy	19.22	2.525	15	24	19	46.3%	22	53.7%
5	Motivation	19.56	2.933	13	25	19	46.3%	22	53.7%
6	Social competence	18.22	2.911	13	25	15	36.6%	26	63.4%

The study showed a non-significant negative correlation between total EI scores and Module-1 scores, but a significant positive correlation with Module-2 scores (rs=0.322*, p<0.05). Module-2 scores have a significant positive correlation with domain scores including Self-control (rs=0.333*, p<0.05), Empathy (rs=0.410**, p<0.05) and Motivation (rs=0.361*, p<0.05) (Table III).

**Table-III:** Correlations of Academic Scores with Total EI and Domain Scores.

EI and Domains	Module-1		Module-2	

	Spearman’s rho	p -value	Spearman’s rho	p -value
Total EI Scores	-0.052	0.745	0.322*	0.040
Self-awareness	-0.063	0.697	0.080	0.621
Self-confidence	-0.148	0.356	0.168	0.293
Self-control	0.192	0.229	0.333*	0.033
Empathy	-0.193	0.226	0.410^**^	0.008
Motivation	-0.020	0.900	0.361*	0.020
Social competence	-0.111	0.490	0.288	0.068

* Correlation is significant at the 0.05 level (2-tailed).

The study showed no significant association of total EI and domain scores with gender (Table IV).

**Table-IV:** Associations of Total EI and Domain Scores with Gender

EI and Domains	Gender				Chi square	p -value

	Male		Female			

	Good	Need Improvement	Good	Need Improvement		
Total EI:	2.4%	14.6%	24.4%	58.55%	0.651	0.411
Self-awareness	2.4%	14.6%	39.0%	43.90%	0.207	0.109
Self-confidence	9.75%	7.31%	34.14%	48.78%	0.679	0.438
Self-control	4.87%	12.19%	29.26%	53.65%	1.000	0.733
Empathy	4.87%	12.19%	41.46%	41.46%	0.419	0.301
Motivation	9.75%	7.31%	36.58%	46.34%	0.685	0.529
Social competence	4.87%	12.19%	31.70%	51.21%	1.000	0.629

Correlation is significant at the 0.05 level.

## DISCUSSION

The study showed that the majority of participants enrolled in three batches of MHPE program of a public sector university, had low EI scores. There is no study to compare these finding in graduates of other MHPE programs, but a study from India, using the SEQSAC, showed that more than 70% medical postgraduates had poor EI scores.^11^ A study from Pakistan also showed similar results in medical and dental postgraduates where majority scored good in the domain of empathy and low in social skills and managing emotions.^12^ The reason could be the lack of EI training in educational programs in the region of South Asia.

Our study showed a significant positive correlation between EI and academic scores with better correlation between EI scores and Module-2 scores compared with that of Module-1 scores. A plausible explanation of this could be that since the program required active engagement and social interaction amongst the students, their non-cognitive social skills developed with the passage of time which affected their performance in Module-2. This observation can be supported by science of learning and development (SoLD) principles that explain the holistic effect of learning environment on the social, emotional and cognitive development of students for their healthy development and academic progress.^13^ The data from the study may assist the MHPE curriculum committee to include a component of emotional skills development as part of the curriculum.

Recent research reveals the importance of non-academic abilities like interpersonal or social skills, in addition to academic abilities for selection of candidates in high stake areas of medicine, like residency programs by using situational judgement tests.^14^ As EI skills is essential for better performance, assessment of these skills could be a part of candidates’ selection for MHPE program.

Our study showed no significant association between total EI and domain scores and gender. The same results were found in studies from India^15^ and Japan.^16^ Contrary result was reported from Australia.^17^ that showed an association of EI with gender.

A number of factors have been reported to affect the EI scores. This study found age and marriage related to good EI scores. Age is reported to affect EI, which increases from the late teens to 40 years and after 50, it slightly decreases or remains stable.^18^ EI is a construct that can be improved with learning and training and does not remain constant throughout life. A study among Pakistan and Afghanistan university students also showed a positive relation between EI and age.^19^ However, a multi-institutional study from the UK, Ireland, Australia and Hong Kong showed no association between age and EI scores.^20^ This could be due to the use of the trait EI questionnaire as the trait EI model suggests that EI remains relatively stable over the lifetime.

Research study from Wuhan on front-line nurses in the current Covid pandemic showed that married nurses had high EI than single nurses, similar to the finding of this study.^21^ As married people have to develop a balance between personal and professional life, it is expected that they have a more control on their emotions and they know how to deal with others for a successful life and can solve their personal and social problems more effectively.

A teacher whether as a clinician or a non-clinician, with social and emotional competencies, develops her/himself as a professional role model and mentor for students, an effective leader for her/his team and institution, an empathetic physician and a medical educationist. The study is the first to address and highlight the importance of EI in MHPE graduates. Considering the study results, improving this construct in graduates may lead to their improved workplace performance and decrease burn out by managing their stress.^22^ Also good empathy and communication skills in clinicians, that are considered as EI skills, may lead to improved patient satisfaction.^23^

### Limitations

It included a smaller number of male students, the inclusion of only one university, and not including experience as a factor affecting EI scores. These may affect the validity of our study and generalizability. As this is an initial study on the topic, the main purpose was to see the correlation between EI and academic scores in the selected sample. Further research on the topic including larger sample size from MHPE programs offered by different universities would be helpful in generalizability of the results.

## CONCLUSION

The study showed a significant correlation between EI and academic scores. It will be interesting to study the performance of the participants of this study in their roles as medical educationists and determine the correlation of their total EI scores and domain scores with the assessment of their performance at the workplace.
